# Farmers’ Perceptions and Drivers of Antimicrobial Use and Abuse in Commercial Pig Production, Ogun State, Nigeria

**DOI:** 10.3390/ijerph17103579

**Published:** 2020-05-20

**Authors:** Oluwawemimo Oluseun Adebowale, Folashade Adefunke Adeyemo, Noah Bankole, Mary Olasoju, Hezekiah Kehinde Adesokan, Olubunmi Fasanmi, Olanike Adeyemo, Olajoju Awoyomi, Olugbenga Kehinde, Folorunso Oludayo Fasina

**Affiliations:** 1 Department of Veterinary Public Health and Preventive Medicine, Federal University of Agriculture Abeokuta, Abeokuta 110124, Ogun State, Nigeria; foladefunke@gmail.com (F.A.A.); maryvet2006@yahoo.com (M.O.); jojuawoyomi@yahoo.com (O.A.); gbengakehinde210@yahoo.com (O.K.); 2 Department of Veterinary Microbiology and Parasitology, Federal University of Agriculture Abeokuta, Abeokuta 110124, Ogun State, Nigeria; noahbankole95@gmail.com; 3 Department of Veterinary Public Health and Preventive Medicine, University of Ibadan, Ibadan 200284, Oyo State, Nigeria; greaterglory2008@gmail.com (H.K.A.); olanikeadeyemo@hotmail.com (O.A.); 4 Department of Veterinary Laboratory Technology, Federal College of Animal Health and Production Technology, PMB 5029, Ibadan 200262, Oyo State, Nigeria; bumaetal@gmail.com; 5 ECTAD, Food and Agriculture Organization of the United Nations (FAO), Dar es Salaam 14111, Tanzania; daydupe2003@yahoo.co.uk; 6 Department of Veterinary Tropical Diseases, University of Pretoria, Pretoria 0110, South Africa

**Keywords:** pig farmers, perceptions, drivers/risk factors, antimicrobial use, antimicrobial stewardship

## Abstract

Antimicrobial resistance (AMR) in humans has been linked to non-judicious antimicrobial use (AMU) in food animals. To develop antimicrobial stewardship plans (AMSPs) for pig farmers, there is the need to understand the current status of AMU and the driving factors in the industry. Data on AMU, farmers’ perceptions of associated drivers, and biosecurity were collected through a mixed-method study design with focus group discussions (FGDs) and questionnaire-based interviews. Antimicrobials (AMs) were mainly used for therapeutic and prophylactic purposes. Common AMs used were tetracycline (78.8%), gentamycin (53.8%), and tylosin (52.5%). Perceived drivers of AMU were linked to economic benefits, farmers’ previous experiences, sick animals, expensive veterinary services, easy accessibility to over-the-counter drugs, poor farm practices, and poor disease prevention strategies. AMU was poor (average 40.2%), while knowledge on AMs and implications for animal and human health was considered averagely satisfactory (56.4%). The biosecurity level was also satisfactory (53.0%) and significantly associated with having a written farm health plan (*p* = 0.035). Good AMU was found to be strongly associated with farmers’ use of veterinary services (*p* = 0.001). Diverse factors drive antimicrobial use among pig farmers in Ogun State, and these could be addressed by providing continuing education on antimicrobial stewardship and best farm practices.

## 1. Introduction

The agricultural sector accounts for approximately 36% of the total Nigerian gross domestic product (GDP), with less than 5% represented by the livestock sub-sector [1]. Although the country’s livestock industry has marginally increased in the last decade, the annual growth rates are still too low to satisfy the animal protein requirements of the increasing human population of over 170 million [2]. The situation has generated persistent malnutrition and low intake of good quality animal protein. Individual protein dietary intake is less than 9 kg in Nigeria, well below the United Nation’s Food and Agricultural Organization’s (FAO) recommendation of 41.9 kg/person. This continues to be a significant public health concern in the country, especially in children [3,4].

In Nigeria, the pig sector of the livestock industry remains underdeveloped compared to the poultry or cattle sectors. Pig production offers an opportunity to contribute to meeting the key sustainable development goals of ending poverty and hunger and promoting good health and well-being [5]. This is because of the unique characteristics of pigs over other food animal species [6], including a fast growth rate and high feed conversion efficiency [6,7,8,9,10]. However, despite high potential, in pig production there are diverse limitations, including inadequate strategies and programs for animal disease prevention and control, poor hygiene and management practices, high mortality rates, and minimal or absent healthcare [6,11]. As a result, abuse of antimicrobials (AMs) by pig farmers occurs as an alternative disease preventive measure, oftentimes without veterinary consultation and prescriptions. Such indiscriminate use of AMs in livestock portends a threat to human health globally because most of the antimicrobials involved are also used for controlling human infections [12].

Through the misuse of antimicrobials (AMs) in food animals, including but not limited to pigs, certain bacterial organisms have developed resistance to antibiotics used in humans, or residues in consumed food of animal origin or the environment [13,14]. The World Health Organization (WHO) has stated that the risk for the emergence and spread of antimicrobial-resistant (AMR) bacteria will intensify due to the irrational use of antibiotics for therapeutic, prophylactic, anaphylactic, and growth promotion purposes in livestock production. Another concern relates to the recent reports of high morbidity and growing mortalities associated with the reduction in the effectiveness of standard life-saving AM drugs used for the treatment of food animal diseases and especially human infections [5,14]. While AMR continues to represent a challenge, the pipeline for new AMs is running dry [15].

To address the challenges above, two main approaches have been suggested: (1) investment in the discovery of new antibiotics and (2) improved global antimicrobial stewardship [16]. In line with these suggestions, the Global and National Action Plans on AMR have been developed with the following priorities: (1) To improve awareness and understanding of antimicrobial resistance; (2) to strengthen knowledge through surveillance and research; (3) to reduce the incidence of infection; (4) to optimize the use of antimicrobial agents; and (5) to develop the economic case for sustainable investment that meets the needs of all countries. Similarly, the Global Health Agenda (GHSA) Antimicrobial Resistance Action Package (GHSA Action Package Prevent-1) has set a five-year target to support the WHO, FAO, and the World organisation for animal health (OIE). This involves the development of an integrated set of activities to combat antimicrobial resistance in humans, animals, agriculture, food, and the environment. In the food and agricultural sector, the FAO identified critical areas needing intervention, including the need to tackle the problem in food animals. This involves capacity development for surveillance and monitoring of AMR and antimicrobial use (AMU), awareness and promotion of ethical farm practices, and the prudent use of antimicrobials in food animals, among others.

To develop efficient educational programs and guidelines on antimicrobial stewardship (AMS) among pig farmers and to design strategic plans, initiatives, and policies that are practical and focused, baseline data which elucidate the current situation are required. We therefore utilized a combination of focus group discussions (FGDs) and a survey questionnaire to explore farmers’ perceptions, the pattern of AMU, and associated drivers in pig production in Ogun State, Nigeria, to generate reference data and provide insights into the current status of AMU in pigs in Nigerian farms and its implications. To the best of our knowledge, this is the first study to explore the drivers for AMU in pig farming in Ogun State. This study may be used as a baseline source for the development of effective antimicrobial stewardship and best farm practices intervention programs to mitigate inappropriate AMU, which is crucial in Nigeria.

## 2. Materials and Methods

### 2.1. Study Location

**Figure 1:**
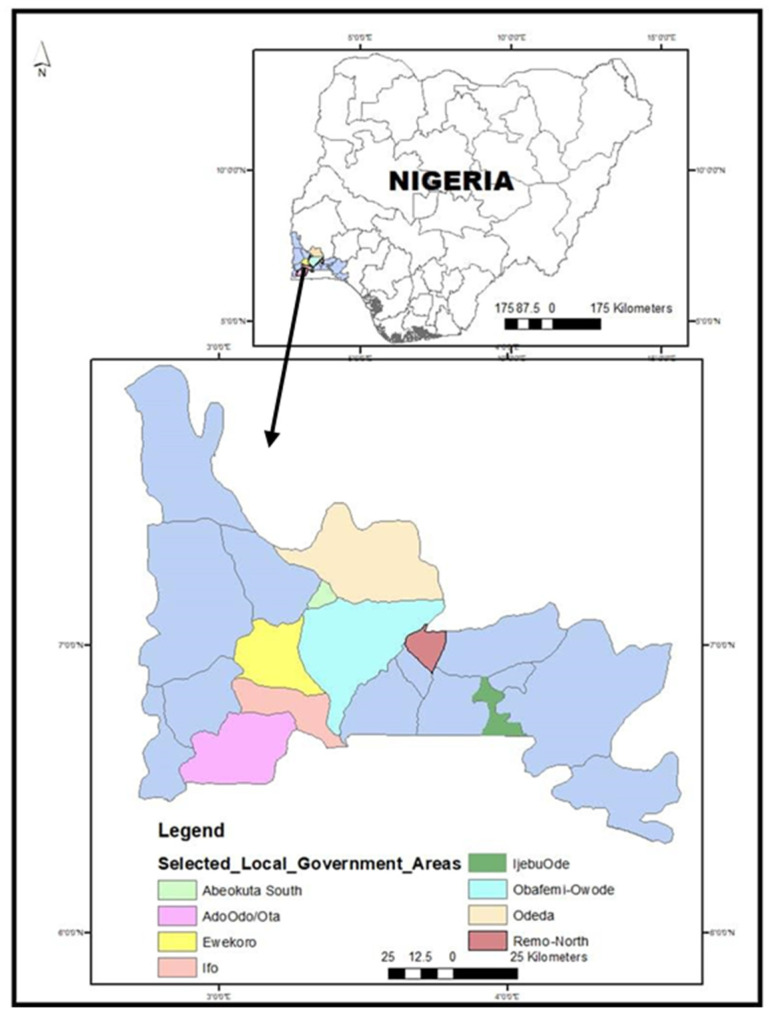
Spatial distribution of local government areas covered in Ogun State.

The study location is Ogun State, South-West Nigeria (latitude: 7°3.5′–9°12′ N; longitudes: 3°35′–5°27′ E). This location borders the states of Lagos to the south, Oyo and Osun to the north, Ondo to the east, and the Republic of Benin to the west. Ogun, with 20 local government areas, has an estimated population of 3,751,140 [17] (Figure 1). The state was selected based on the relatively high livestock production activity, including pig, catfish, and poultry production [18]. Furthermore, Ogun State has one of the densest pig populations in Nigeria [18].

### 2.2. Study Design, Sample Size, and Recruitment of Pig Farm

Using mixed methods, a combination of focus group discussions (FGDs) and survey questionnaires was implemented. Phase 1 included focus group discussions to understand the perceptions of farmers relating to antimicrobial usage and practices, challenges in pig production in Ogun State, Nigeria, and recommendations on making AMs work at the various stakeholder levels of the industry. Phase 2 involved the administration of questionnaire-based interview in a cross-sectional study to describe the characteristics and profiles of pig farms and owners in the state, including the risk factors or practices for compliance with biosecurity, level of antimicrobial use, knowledge of AMs, and perceived side effects among pig farmers.

For the FGDs and the cross-sectional survey (interviews), all pig farms registered under the Pig Farmers’ Association were identified. The Chairman of the Ogun State chapter of the Pig Farmers Association of Nigeria (PFAN) was contacted via email and followed up with telephone calls. Signed consent for the Association to participate in the study was obtained from the Chairperson (S1) on behalf of the group, and individual verbal consent from farmers registered was obtained. Participation was based on individual availability and willingness to participate in the study, and everyone was notified of his/her right to discontinue participation at any stage of the study [19].

#### 2.2.1. Focus Group Procedure and Structure

The FGDs were conducted during the Association’s monthly meetings in August 2019. Before the study, a briefing on the concept of the project was provided by the moderator, and consent to participate was sought verbally. All participants (*n* = 45) were individually assigned an identification number to maintain anonymity and for the purpose of random mixing of the group. The group was then purposively divided randomly into three groups comprising 15 consenting participants per group. The inclusion criteria included (1) mandatory registration with the Ogun state chapter of the PFAN, and (2) ownership of and management of pigs in the last 12 months. Each FGD lasted approximately 120 min. Three out of the four researchers conducting the study served as moderators in each of the groups. A panel of pretested interview guides composed of 14 open-ended questions was used for the FGD (S2). The moderator’s roles and responsibilities were to guide the discussion, ask follow up questions, confirm that all areas in the topic guide were addressed, ensure that no individual was prevented from expressing his/her opinion, and make certain that the moderator did not influence the process with his/her own opinion [20]. Participants were prodded to explore the topics in depth, reflect on discussions, and raise their issues. Handwritten notes of essential points and audio recordings of the interviews were taken following consent from the participants. The audio recordings were transcribed for clarification on the key points and analysis.

#### 2.2.2. Cross-Sectional Study and Farm Recruitment

A total of 98 farms were identified for the cross-sectional survey. The sample size for a simple random sampling was calculated using Win Epi 2.0^®^ software for percentage frequency in the population. The following assumptions were used: finite population because the target population was drawn from lists of registered farmers, 50% expected frequency of respondents that used antimicrobials in pig farming, a desired absolute precision set at 5%, and a 95% confidence interval. Based on these assumptions, a sample size of 78 pig farmers/farms was needed.

#### 2.2.3. Questionnaire Design, Pretesting, and Data Collection

Through a review of literature on predictors and risk factors, practices, and knowledge of farmers on AMU as well as qualitative experts’ opinions, a set of closed-ended questions was designed to ease data processing and improve response precision for the cross-sectional survey [21]. The questionnaire (S3) was divided into five sections, viz: (1) pig farms and farmers’ demographic characteristics (16 questions); (2) biosecurity and management information (20 questions); (3) farm health records (7 questions); (4) AMU practice in pigs (12 questions); and (5) knowledge about antimicrobials and public health implications (13 questions). The questionnaires were pretested among 34 pig farmers [22] in a settlement before the final administration (test for reliability ≥ 0.7) to identify problems (if any) and adjust the questionnaire for adequate data capture.

### 2.3. Data Management and Statistical Analysis

The focus group discussion responses (handwritten notes and audio recordings transcribed verbatim) were analyzed by researchers following several meetings and outcomes to capture all the details. Survey responses were first captured in and summarized in a Microsoft Excel^®^ 2016 spreadsheet (Microsoft Corporation, Redmond, WA, USA). Descriptive statistics were calculated for all variables in the forms of frequencies and proportions. To estimate the levels of biosecurity, AMU, and knowledge about AMs and public health effects among pig farmers, binary responses were captured as follows: “Yes” and accurate responses were scored “1”, and “No” and inaccurate responses were scored as “0”. The scoring system for AMU and biosecurity levels ranged between 0 and 15, while that for knowledge was between 0 and 13. All scores were converted to 100%. The cumulative score range was further re-categorized as “poor” (≤50%), and “satisfactory” (>50%). Associations between variables were determined by binary logistic regression analysis (BLRA) using STATA 12.0. The binary logistic regression was used to determine the association or impact of pig farmers’ sociodemographic profiles with regard to biosecurity levels, antimicrobial use/practices, and knowledge. For the BLRA, *p* < 0.05 was considered statistically significant at a probability cut-off of 0.05. Odds ratios (OR) were computed to determine the strength of associations between variables at 95% confidence intervals (CIs).

## 3. Results

### Demographic Characteristics of Participants

#### 3.1.1. Focus Group Participants’ Demographics/Characteristics

A total of 45 pig farmers (8 females and 37 males) from across the various local government areas of Ogun State participated in the three FGDs. The participants’ ages ranged from 25 to 75 years. All participants were involved in pig production, and did not self-identify as organic pig producers.

- Pig farmers’ perceptions about antimicrobial use and practices

**Figure 2:**
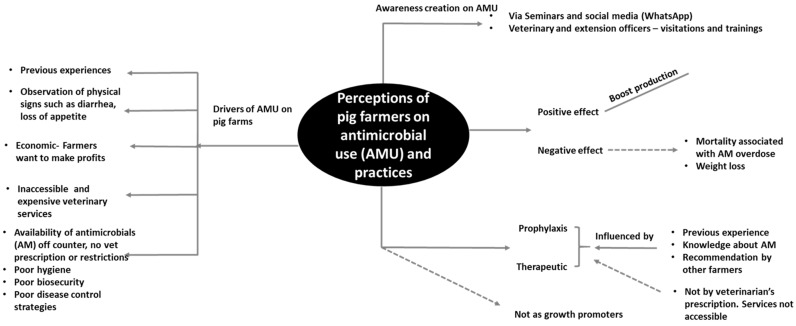
A thematic map showing perceptions of pig farmers that participated in the focus group discussions (FGDs) on antimicrobial use (AMU) and practices, drivers for overdependence on AMU, and challenges confronting pig production in Ogun State. AM: antimicrobial.

Figure 2 summarizes participants’ perceptions on AMU and practices, drivers for overdependence on AMs, challenges confronting the pig industry in Ogun State, and diverse recommendations on contributions of stakeholders in ensuring efficient use of AMs.

Antimicrobial use was perceived by farmers to positively boost pig production, but occasionally things could go wrong when there was erroneous drug administration (especially overdosage). Detailed descriptions and excerpts of the participants on their perception of the effect of AM on pig production are captured in Box 1.

Box 1 **Farmers’ opinion on antimicrobial usage.**

*“[…...] It (usage of antibiotics) is both sides, either positively or negatively on production. When an animal is sick, for example in the case of a pregnant animal that is sick and you use antibiotics on it, the fetus will go down, there will be premature farrowing and apart from that, the animal loses weight [.……]” …… FG 3*

*“We are also very careful when it comes to dosing them with antibiotics because we don’t want to lose them (pigs). The first time I injected them, I observed they were going down and I had to go and take a refresher course on drug administration” …… FG 1*

*“When pigs go down like that, what I do is to press their heart severally and they jump back to life. What we need from veterinarians is to train us on proper dose administration, and resuscitation techniques so that we don’t lose our animals” …… FG 1*

*“I prefer to use herbs because it has limited or no side effects, all antibiotics have adverse side effects because they are synthetic unlike herbs that are natural” …… FG 2*

Farmers commonly used AMs for prophylaxis and therapeutic purposes but denied using them as growth promoters; however, they confirmed administering them in animal feeds without the recommendation of professional veterinarians. The preferred route of administration was parenteral (injection). Based on perceptions, the most commonly used AMs by pig farmers in the state were penicillin, streptomycin, gentamycin, and long-acting (LA) oxytetracyclines (20%), with the latter being used to treat all disease conditions. Nevertheless, based on their experiences, they were aware of the specific AMs to use for specific diseases. In terms of prescription and drug administration, the majority of FG participants personally medicated their animals and occasionally consulted with veterinarians. Most producers relied on their own experience, knowledge, judgement, and recommendations from other farmers when deciding to administer antimicrobials to pigs. However, in difficult situations, they consulted veterinarians. Other identified factors that influenced farmers’ decisions to use AMs were cost, the composition or the active ingredient, and efficacy, but not the drug producers or brand names (Box 2).

Box 2 **Farmers’ confirmation on abuse of antimicrobials. LA: long-acting.**

*“Infections will manifest and there are drugs you can use. With broad-spectrum like LA oxytetracyclines (20%) and the indications for use written clearly on the bottle including dosage, diseases, and withdrawal period, so you administer if it’s going to be once let it be once and if twice and if you see the disease is coming down then you give another shot, then it’s off. Most of us have used LA before to treat our sick animals and it works. But, when animals do not recover, we sell them off but none of us observes the withdrawal period”.…… FG 3*

*“We are confessing to you that we treat our own animals. I am my own vet. I diagnose and treat my animals because I don’t have access to veterinary services” …… FG 1*

*“Most knowledge of AM use we have (to date), are not from veterinarians but have been from past experiences, Internet, and recommendations from other farmers with similar cases. Once a drug is effective, we keep buying” …… FG2*

- Perceptions of drivers of farmers’ dependence on antimicrobials

The major factors driving farmers’ use of AM were: (1) animal discomfort and physical signs such as diarrhea, coughing, mange, loss of appetite; (2) farmers’ previous experiences; (3) availability and accessibility of AM off-counter veterinary outlets without veterinary prescriptions or restrictions; (4) inaccessibility to expensive veterinary services; (5) poor farm hygiene and management practices; (6) poor disease control and management strategies; and (7) economic efforts to make a profit. Farmers stated that the usage of AMs was halted as soon as an animal was active or back in good condition, and sometimes according to the drug manufacturer’s instructions. Participants linked the occurrence of AMR to the overuse of AMs and lack of alternatives. A farmer opined on what influenced them to commence and stop using AM as follows: “The state of the animal, whether the sickness is mild or the animal is seriously sick. The disease conditions influence the use of antibiotics, when you notice your animal is sick you give antibiotics and when the animal is well, you discontinue the use.” (FG 2).

- Perceptions relating to challenges confronting the pig industry in Ogun State

The participants listed and discussed various constraints and major challenges which were experienced by pig farmers in Ogun State, including the following: (1) lack of and high cost of quality feeds; (2) diseases and poor preventive or control strategies; (3) poor accessibility to veterinary services and extension officers; (4) substandard and fake drugs; (5) poor market for pigs and their products; and (6) poor infrastructure, especially roads and clean water. The farmers confirmed that no funding facilities (loans, grant, and government support) had been provided for pig farming in the country. Specific responses are captured in Box 3. Similarly, farmers expressed their displeasure about the status of veterinary extension services in the country (Box 3).

Box 3 **Farmers’ opinion and frustration on the challenges of the pig sector.**

*“The government doesn’t even give us any money. There is a clear bias for cattle producers when it comes to funding. We fill applications forms and submit same to the appropriate headquarters, a body which is usually chaired by person who is aversed to pig rearing, hence we don’t get any funding. It is like some religions are against the expansion of pig farming in the state” …… FG 1*

*“Many common diseases and conditions that challenge pig health include but are not limited to mange, foot rot, helminthiasis, diarrhoea, anestrus, Africa swine fever, pneumonia, abortion/stillbirth and piglet anaemia. These diseases or conditions increased more whenever there was a drop in temperature” ……. FG 1*

*“We have zero inputs. Veterinary extension services should go round. They should visit farms and tell us what services they can offer us. They don’t show up. Vet extension has gone to extinction” ……. FG 1*

*“The vets are the intermediaries between the producers and the government and so they should rise up to their responsibilities” ……. FG 3*

- Proposed solutions to ensure effective use of AMs by respondents

The interviewed stakeholders provided recommendations as follows:

1. Pig producer level: The farmers suggested that improvements in best practices in animal management (e.g., housing, quality feeding, and alternatives to antibiotics), hygiene, and biosecurity should be given priority, but did not indicate who should bear the cost of this improvement. The need to acquire adequate knowledge on AM stewardship and effects on animal and human health was considered important by all participants.
2. Veterinarian level: The farmers recommended that veterinarians should make their services available and affordable, and requested that proper diagnosis of diseases be ensured before drug prescription, especially AMs. Furthermore, they indicated that the development of research innovations for alternative medicine (e.g., herbal therapy) and pig management techniques (e.g., organic farming) should be intensified. Participants wanted more practically oriented training and workshops on prudent AMU and animal management to improve their AMU in pig production (Box 4).
3. Government level: The participants suggested that the government should enforce quality control of feeds and drug production, and competitive importation should be taken seriously. The participants also wanted the authorities to identify with pig farmers and support the provision of loans, while enforcing policies to enhance pig operations in the country, fund veterinary and extension services adequately, and employ more veterinarians to cover all the local government areas of the state. The farmers suggested that an AM stewardship campaign and education be channeled through seminars and regular media. The participants confirmed that the FGD was educative and informative with respect to knowledge on antibiotic use, and that the understanding of prudent use would make them work more efficiently for the sustainability of the livestock industry.

Box 4 **Farmers’ expectations from professionals.**

*“Henceforth, we don’t want abstract training. We are practical people; we are hands-on. Most of the training they provide is abstracts. Not everyone is educated but if you show them the technical know-how, they will practice them on their farms” …… FG 1*

*“The veterinarians should educate us on pig diseases, physical recognition and manifestations of these diseases. We pick information randomly from books or Internet” …… FG 3*

#### 3.1.2. Survey Participants’ Socio-Demographic Characteristics

Table 1 describes the demographic profile of the farmers. A total of 88 pig farmers participated in this survey. The majority of respondents were male (70/88, 79.5%) and married (71/88, 80.7%), while 17.0% (15/88) were single and 2.3% were divorced. The median age of participants was 45 years (minimum 22, maximum 81). Most participants had received tertiary-level education (73/87, 83.9%), while 1.1% (1/87) and 14.9% (13/87) had a diploma certificate or secondary school-level education, respectively. Pig farming was observed to be the main livelihood for 58.6% (50/88) of the participants, while others had secondary careers such as business, arable farming, teaching, banking, accounting, government work, and estate management. A high proportion of farmers (63/87, 72.1%) worked under 6 h a day with the pigs, with the remainder working over 6 h.

#### 3.1.3. Survey Farm Characteristics, Management and Biosecurity Practices

Table 2 describes the various farm characteristics and management practices of pig farms in Ogun State. Over half (40/71, 53.3%) of the farms surveyed had been in operation for over 6 years (median 6 years, minimum 6 months, maximum 30 years), and farm sizes ranged from a minimum of 1 plot to a maximum of 60 plots (median 1). Standard plots measured an average of 648 square meters. The minimum and maximum numbers of enclosures recorded were 1 and 160 (median 13), and the total numbers of male and female animals present were 584 (median 4) and 1478 (median 16), respectively. The mean ages of adult and young pigs were 4.5 years (±3.8, 95% CI 2.1–7.0) and 3.4 months (±1.2, 95% CI 2.5–4.3), respectively.

Some of the farmers (15/88, 17.0%) practiced mixed farming i.e., other animals aside pigs were present. For example, sheep were kept for commercial purposes, goats for breeding, and poultry in particular for egg production. The majority of pig farms had exotic breeds and their crosses (66.7%), especially the Large White, Landrace, Hampshire, and Duroc breeds, in that descending order, and few had indigenous pigs. Most of the farmers (91.9%) sourced or purchased pigs from other farms within and around Ogun State, such as farms from neighboring states including Ibadan in Oyo State. Pigs were kept for fattening (64.9%) and breeding (22.8%) purposes. The respondents practiced closed housing (72/84, 85.7%) whereby animals were kept in pens or enclosures with gates, and 60/88 (68.1%) had at least one enclosure separate for boars, sows, weaners, or growers. The farms investigated employed at least one farm attendant (median 1, minimum 1, maximum 4; Table 2), and the most common production system in Ogun State was intensive farming (46/74, 62.2%).

Table 3 describes the biosecurity practices of pig farms in Ogun State. In terms of biosecurity, 85.2% of respondents were aware of this concept. Over 70% of respondents indicated they did not observe movement restrictions as they visited other farms, and allowed feed transport vehicles and buyers have access to their farms. Meanwhile, 79.6% of the farmers reported that they had their own farm equipment and avoided borrowing items from other farms, and also performed regular cleaning and disinfection (93.2%). The most common forms of waste disposal on pig farms were open dumps and bury/burn methods. Overall, at the farm level biosecurity was averagely satisfactory (53.0%). With respect to the biosecurity levels of respondents, about 43.2% fell within the poor category, while others were satisfactory (56.8%). The presence of a written farm health plan was found to be strongly associated with good biosecurity. Farms without a written health plan had lower odds (OR = 0.39; 95% CI = 0.16–0.94; *p* = 0.035) of observing good biosecurity than those with a written health plan (Table 4).

#### 3.1.4. Farm Health and Antimicrobial use Practices

**Figure 3:**
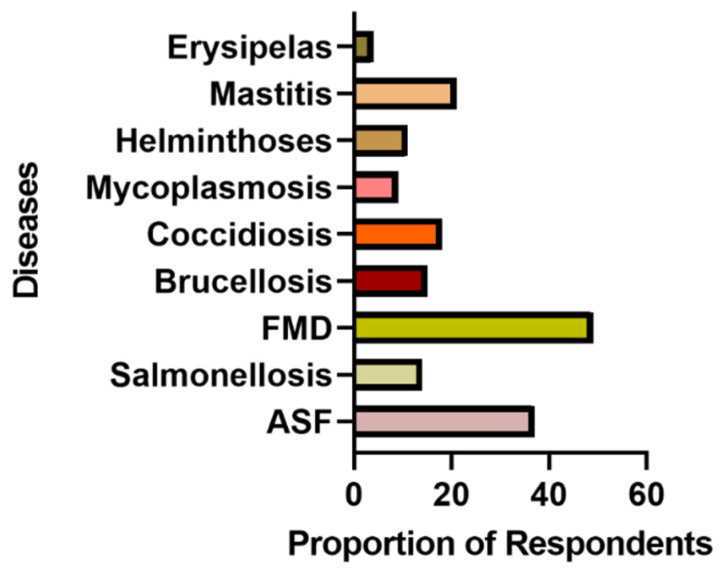
Common diseases self–reported by respondents during the survey. FMD: foot-and-mouth disease; ASF: African swine fever.

With respect to farm health, 54/87, 52/88, and 50/88 reported having a written health plan, keeping farm records, and making consultations with veterinarians, respectively. Foot-and-mouth disease (49/73, FMD), African swine fever (37/72, ASF), mastitis (21/71), coccidiosis (18/72), and brucellosis (15/72) were the five most common diseases reported by pig farmers and for which AMs were used (Figure 3).

**Figure 4:**
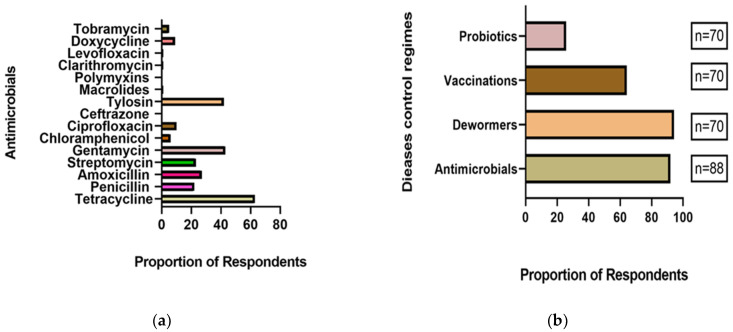
Common antimicrobials used by farmers and disease control regimens. (**a**) Self-reported AMs used by respondents in the treatment of pig diseases; (**b**) Various disease control regimens employed by pig farmers who participated in the survey.

In the survey, AMs were extensively used by 92.1% (81/88) of respondents, of which 53.7% used them often. AMs were used mainly for therapeutic purposes (72.3%), prophylaxis (36.9%), and as growth promoters (29.2%), in contrast to responses in the FGD. The main route of administration of AMs was by injection (intramuscular, 95%), while topical application was employed the least (1.2%). In increasing order, penicillin, streptomycin, amoxicillin, tylosin, gentamycin, and tetracycline were mentioned by the survey participants as being commonly used (Figure 4a). The disease control regimens employed in the farm varied in proportions (Figure 4b). The AMs were given by the farmers themselves (64/88; 72.7%) and were mainly purchased mainly over-the-counter ( 58/88; 65.9%). In terms of whether company brands influenced the choice of AMs, over half (59. 2%) reported they did not.

The overall farm practice level of AMU was estimated to be poor (40.2%). Regarding the practice levels of individual respondents, almost three-quarters (73.9%) engaged in poor practices, while others fell within the satisfactory category (26.1%). The educational status of farmers was positively associated with AMU practices. Specifically, farmers without high educational qualifications (secondary school qualifications and below) had reduced odds (OR = 0.18; 95% CI = 0.02–1.48; *p* = 0.111) of compliance with good AMU as compared to those with educational qualifications above the secondary school certificate level. On the other hand, employing the services of veterinarians was statistically associated with good practices of AMU. Farmers who did not use the services of veterinarians were about 13 times (OR = 0.076; 95% CI = 0.02–0.35; *p* = 0.001) less likely to have good AMU practices than those who employed the services of veterinarians (Table 4).

The general knowledge of AMs and side effects in animal and human health was determined to be moderately satisfactory (56.4%). At the individual level, 35.2% and 64.8% displayed poor and satisfactory knowledge, respectively. For instance, a low number of the respondents (19/88) believed it was safe to complete AMs even after animals had recovered, while less than half (39/88) indicated a need for AM sensitivity tests before their use or administration. On the other hand, a higher proportion of respondents understood that AMR increased morbidity and mortality in animals (59/88) unlike in humans (33/88). In relation to AM stewardship, approximately three-quarters (67/88, 76.1%) had no knowledge of the concept, and only 1 (4.7%) of the 21 (23.9%) participants who were aware of the concept could correctly explain its meaning. Farmers’ levels of knowledge were associated with pig production systems. Interestingly, pig farmers involved in semi-intensive production were 2.8 times (OR = 2.87; *p* = 0.037; 95% CI 1.07–7.74) more likely to have good knowledge of AMs and side effects than farmers involved in intensive farming (Table 4). Concerning gender, female were three times more likely to have better knowledge of AMs and side effects than males (OR = 3.06; 95% CI 0.82–11.47; *p* = 0.09). Conversely, farmers older than 45 years old had reduced odds of knowledge on AMs as compared with those below this age (OR = 0.43; 95% CI 0.16–1.15; *p* = 0.093).

## 4. Discussion

Understanding the current patterns of AMU in livestock is important in order to develop strategies for optimal antibiotic use in order to slow down the emergence of AMR in animal production [23]. It has been documented that half of the world’s antimicrobial production is used in farm animals [24]. Globally, livestock farmers use AMs as growth promoters (with the possible exception of Europe where this has been banned since 2006) and for therapeutic and prophylactic purposes [24]. For therapy, AMs are generally used at high doses for short periods of time on individual animals in order to treat specific diseases. For prophylaxis, they are generally administered at high doses for short periods of time (for example after weaning or during transport) to groups of animals in order to prevent the occurrence of specific diseases. For growth promotion, AMs are also used for groups of animals but at a low doses and for long periods of time in order to reduce bacterial competition in the gut and improve feed conversion.

Misuse and abuse of AMs in livestock represent a major cause for concern due to the potential for AMR [25,26,27]. AMR has recently gained worldwide recognition because the emergence of multidrug-resistant organisms has led to increased mortality and a huge economic burden [28,29]. Due to the fact that Nigeria is no exception to the challenges of non-judicious use of AMs and AMR, there is a need for a detailed understanding of factors that influence livestock producers’ decision-making, their beliefs, attitudes, and perceptions as a basis for building effective intervention strategies in curbing the burden of over-dependence on AMs and associated AMR [30]. Hence, identifying pig producers’ current perceptions, knowledge, and drivers of AMU is a critical step towards achieving success in policy interventions and education/awareness that promote judicious AMU. This study provides an understanding of pig farmers’ perceptions of factors driving the use of AMs and alternative treatments to AMs, appropriate avenues for disseminating information on prudent AMU, and the challenges that pig producers encounter the country. We also reported on the biosecurity status and prevalence of antimicrobial usage and associated drivers of risk factors in pig farming in parts of Nigeria.

Numerous factors and drivers of AMU identified in this study have been corroborated in other studies [20,31,32,33], and have been shown to increase susceptibility to a wide range of diseases [6,11]. The high costs of pig production in terms of feeding, management, and disease control are major economic factors that drive farmers to use poorer quality feeds (which can potentially carry pathogens) and abuse AMs, especially as growth promoters and for prophylactic use to enhance productivity.

The important drivers reported by farmers in these studies (FGD, and survey) were: (1) the ability of livestock farmers’ to access AMs as “over-the-counter drugs” without a requirement for a prescription from a veterinarian; (2) their previous experience; and (3) poor management and biosecurity practices. Administration of AMs by the farmers to their animals possibly arose from farmers’ dissatisfaction with high cost of veterinary services and poor access. Medication of animals through the over-the-counter acquisition of AMs by livestock producers has been reported in Nigeria [5,32,34,35], and such reports have suggested the need for the enforcement of appropriate regulatory policies to prevent irrational use of drugs, for example by prohibiting antibiotic sales without doctor’s prescriptions. A predisposition to rely on personal experience, as mentioned in this study, sometimes leads farmers to use drugs indiscriminately and makes them oblivious of the need to consult veterinarians, whose services are subsequently required when all obtainable means of treatment have failed. Furthermore, a lack of hygiene and sanitary measures, inadequacies in management and animal husbandry practices, and poor adherence to biosecurity practices contribute to the high use of antibiotics for the prevention of disease outbreaks [32].

We reported an average biosecurity score of 53% on pig farms, but we are of the opinion that the usage of veterinary expertise and regular consultation in developing specific biosecurity programs will mitigate over-dependence on AMs. In the European Union, empirical evidence has confirmed that the implementation of farm health plans and the utilization of veterinary services reduces AMU in food animals [36,37,38]. Similar efforts should benefit pig farmers in Ogun state. A study earlier reported that good educational qualifications improved knowledge on antimicrobials and their use, and we arrived at a similar conclusion [39]. The level of adherence to good practices in AMU in this study was poor (40.2%). This could have resulted from the lack of awareness on antimicrobial stewardship (AMS), as well as poor implementation of prudent AM usage. Veterinarians play important roles in animal health and antibiotic stewardship and often farmers rely on veterinarian’s advice on pig health, choices, and use of antibiotics [40]. Innovative educational interventions on AMS among livestock producers through veterinary expertise in Nigeria require urgent attention and implementation. In the present survey, less than 30% of farmers claimed to be aware of the concept of “antimicrobial stewardship” and only one could explain what it meant. The respondents from the FGD requested that training on AMS be practical, and the preferred means of communication on AMS were seminars and social media platforms such as WhatsApp^®^. Innovative research on alternative treatments or complementary therapy such as herbal remedies and improved animal husbandry techniques including organic farming to assist in the reduction of AMU in the pig industry was requested.

Antimicrobials are used for therapeutic, prophylactic, and growth promotion purposes in pigs, and therapeutic use of antimicrobials was most commonly reported in this study. Farmers use AM to treat sick or critically injured pigs and to prevent/control disease spread, usually based on farmers’ previous experiences and recommendations from other farmers. In Vietnam, South East Asia, over 30 different AMs are used for therapeutic purposes [41]. It has been established that the term “growth promoters” is sometimes used to qualify AMs aiming at disease prevention [42], since antimicrobials are often used in low doses in feed to improve growth rate by destroying and inhibiting bacteria. Consequently, low dosages may lead to the resistance of bacteria to antimicrobials. All European countries have banned the usage of antibiotics for growth promotion, but these are still utilized in countries like the United States of America and China, although this use is regulated by authorities [41,43,44]. Despite their ban in many countries, AMs continue to be used for growth promotion and infection prevention globally [23].

The most commonly used antimicrobials identified in this study included tetracycline, gentamycin, tylosin, amoxicillin, and penicillin, in accordance with previous findings [23,45,46]. These drugs are commonly used by farmers because they are readily available, cheap, and cost-effective as compared with newer-generation antimicrobials. Tetracyclines are some of the cheapest broad-spectrum antimicrobials obtainable over the counter without a veterinary prescription for use in humans and animals. Tetracyclines are commonly used as bacteriostatic drugs effective against rapidly dividing microorganisms. For instance, many respiratory diseases are often managed by using long-acting (LA) oxytetracyclines and tylosin, gentamicin is used to treat inflammatory diseases, and beta-lactams such as penicillins and amoxicillin are used for mastitis, metritis, and erysipelas treatments in pigs. The FDG revealed farmers’ preference for LA oxytetracycline because of its broad bacteriostatic coverage against bacteria. Studies conducted in South Africa [47] and Tanzania [24] similarly confirmed that tetracyclines and beta-lactams are the most often used antibiotics in food animals, as found in our study. However, while oral medication was the most common route of antimicrobial administration in pig production elsewhere [48,49,50,51], our findings contradicted this assertion.

Finally, it was observed that the common diseases for which AMs were used in the survey were similar to those reported by farmers during the FGD. Such diseases included foot-and-mouth disease, African swine fever, mastitis, coccidiosis, and brucellosis, most of which are transboundary animal diseases (TADs) with significant economic, trade, and food safety (antibiotic residue and AMR) implications. For instance, foot-and-mouth disease, one of the most important diseases reported in this study, is endemic in Nigeria, and a highly contagious TAD of great economic significance due to the high morbidity and export restrictions [52,53,54]. The trend of the disease occurrence is unknown or underestimated as there are regular outbreaks, no control measures, and poor disease reporting in Nigeria [55]. It is however surprising to have had a high number of farmers reporting FMD in their pig herds. Cattle are the most susceptible animal to infection by FMD, followed by sheep, while the least susceptible animals are goats and pigs in Nigeria [55]. While some diseases are easier to diagnose because of characteristic clinico-pathological signs like ASF, mastitis, and coccidiosis, others may not have clear-cut symptoms, leading to confusion by farmers. For example, the farmers were unable to distinguish clearly between FMD and footrot, or between brucellosis and other causes of abortion in pigs [56,57]. In a situation of misdiagnosis by farmers based on the above, medication may be wrongly applied, with AMR as an outcome. Part of the training on good husbandry practices should factor in the identification of some of this features to reduce the incidence of misdiagnosis. Such training may also have additional benefits because trained farmers may be used as community disease reporters.

### Feedback Seminar

A feedback seminar on the study outcome was held for pig farmers approximately 6 months after the study was conducted (18 February 2020). Approximately 31% (27/88) of the interviewed farmers attended (6 females and 21 males). The farmers suggested a good working relationship between farmers’ organizations and academia, and confirmed their readiness to collaborate in any field-based or adaptive research that would be beneficial to pig production and sustainability. The farmers strongly affirmed the need to revive extension services, which represent intermediary profession that links research outputs from academia to end-users. The improvement of veterinary services and adequate support from the government are crucial, and farmers advised the government to adequately capacitate local government areas by veterinarians both in terms of number and skills in order to ensure easy accessibility. Regular antimicrobial stewardship training and sessions on good farming practices in pig production were requested. Finally, the farmers requested a multi-stakeholder platform (relevant ministries, academia, farmers, marketers, exporters, and value chain actors) for broader consultation using the One Health approach. This would facilitate brainstorming and the development of plans to improve the sector and promote the export potential of pigs and related products, as desired by pig farmers.

## 5. Conclusions

This study provided insight into factors driving the use of antimicrobials in pig production in parts of Nigeria. Compliance with good practice of AMU was poor, while knowledge on AMs and consequences for public health and biosecurity was moderately satisfactory. The development of farm guidelines, protocols and policies and the promotion of educational training programs on prudent use of antimicrobials are crucial interventions to assist farmers to make the best decisions on antimicrobial resistance and antimicrobial stewardship for pig farmers. Implementation of mandatory farm-specific health plans written in consultation with veterinarians should enhance good biosecurity and husbandry practices on pig farms. Research into alternative therapies to AMs should be prioritized by industry stakeholders. For instance, a research focus on the use of feed supplementation to improve endogenous pig gut defense against harmful pathogens may be needed. Antimicrobial culture and sensitivity testing in clinical practice among pig and other livestock farmers should be promoted.

## Figures and Tables

**Table 1:** Demographic profile of pig farmers in Ogun State, Nigeria.

Variables	Proportion (%)	95% CI
Sociodemographic characteristics of participants		
Gender (*n* = 88)		
Male	70 (79.5)	69.9–86.7
Female	18 (20.5)	13.7–30.1
Marital Status (*n* = 88)		
Married	71 (80.7)	71.1–87.7
Single	15 (17.0)	10.5–26.5
Divorced	2 (2.3)	0.13–8.4
Age (*n* = 64)		
<45 years	31 (48.4)	36.6–60.4
≥45 years	33(51.6)	39.6–63.4
Educational Level (*n* = 87)		
Secondary	13 (14.9)	8.8–24.0
Diploma	1 (1.1)	0.01–6.8
Tertiary	73 (83.9)	74.7–90.3
Working Hours (*n* = 87)		
<6 h	63 (72.4)	62.2–80.8
>6 h	24 (27.6)	19.3–37.8
Employed Farm Attendants (*n* = 88)		
Yes	76 (86.4)	77.5–92.2
No	12 (13.6)	7.8–22.5

*n* = number of responses; CI: confidence interval.

**Table 2:** Farm characteristics and management practices on the studied pig farms in Ogun State, Nigeria.

Variables	Proportion (%)	95% CI
Farm Characteristics		
Farm establishment (in years, *n* = 75)		
<6	35 (46.7)	35.8–57.8
≥6	40 (53.3)	42.2–64.2
Number of male pigs (*n* = 65)		
<4	27 (41.5)	30.4–53.7
≥4	38 (58.5)	46.3–69.7
Number of female pigs (*n* = 67)		
<15	32 (47.8)	36.3–59.5
≥15	35 (52.2)	40.5–63.8
Number of adult pigs (*n* = 41)		
<16	20 (48.8)	34.35–63.5
≥16	21 (51.2)	36.5–65.8
Number of young (*n* = 43)		
<16	20 (46.5)	32.5–61.1
≥16	23 (53.5)	39.0–67.5
Mixed farming (*n* = 88) i.e., other animals		
Yes	15 (17.0)	10.5–26.4
No	73 (83.0)	73.7–89.5
Production system (*n* = 74)		
Intensive	46 (62.2)	50.9–72.57
Semi-intensive	28 (37.8)	27.6–49.2
Type of housing (*n* = 84)		
Open	12 (14.3)	8.21–23.5
Closed	72 (85.7)	76.5–91.8
Enclosure flooring (*n* = 80)		
Soil	0 (0.0)	0.0–0.1
Concrete	80 (100.0)	−100.0
Grass	0 (0.0)	0.0–0.1
Gravel	0 (0.0)	0.0–0.1
Animals permanently held (*n* =88)		
Yes	50 (56.8)	46.4–66.7
No	38 (43.2)	33.3–53.6
Pig breeds present (*n* = 57)		
Exotic	38 (66.7)	53.7–77.6
Local	4 (7.0)	2.3–17.2
Cross	12 (21.1)	12.3–33.4
Exotic and cross	3 (5.3)	1.2–15.0
The purpose of raising pigs (*n* = 57)		
Breeding	13 (22.8)	13.7–35.3
Fattening	37 (64.9)	52.0–76.0
Fattening and breeding	7 (12.3)	5.8–23.6
Pig sources/purchase (*n* = 61)		
Other farms	56 (91.8)	81.9–96.9
Market places	4 (6.6)	2.1–16.2
Other (research institution)	1 (1.6)	0.01–9.5

*n* = number of responses.

**Table 3:** Farm biosecurity on the studied pig farms in Ogun State, Nigeria.

Variables	Proportion (%)	95% CI
Farm Biosecurity Practices		
Awareness of biosecurity (*n* = 88)		
Yes	75 (85.2)	76.2–91.3
No	13 (14.8)	8.7–23.8
Presence of an isolation bay for sick animals (*n* = 88)		
Yes	59 (67.15)	56.7–76.0
No	29 (32.9)	24.0–43.3
Quarantining of new animals on arrival (*n* = 88)		
Yes	52 (59.1)	48.6–68.8
No	36 (40.9)	31.2–51.4
Presence of foot dip at pen entrances (*n* = 88)		
Yes	48 (54.5)	44.2–64.5
No	40 (45.5	35.5–55.8
Visits to other farms by the farm owner (*n* = 88)		
Yes	69 (78.4)	68.6–85.8
No	19 (21.6)	14.2–0.31.4
Visits to other farms by attendants(*n* = 88)		
Yes	53 (60.2)	49.8–69.8
No	35 (39.8)	30.2–50.2
Access to the farm by buyers (*n* = 88)		
Yes	67 (76.1)	66.2–83.9
No	21 (23.9)	16.1–0.33.8
Access to the farm by feed transport vehicles (*n* = 88)		
Yes	64 (72.7)	62.6–81.0
No	24 (27.3)	19.0–37.4
Visits to other farms by the same feed transport vehicles (*n* = 88)		
Yes	70 (79.6)	69.9–86.7
No	18 (20.4)	13.3–30.1
Possession of farm equipment (*n* = 88)		
Yes	70 (79.6)	69.9–86.7
No	18 (20.4)	13.3–30.1
Use of farm equipment from other farms (*n* = 88)		
Yes	10 (11.4)	6.1–19.8
No	78 (88.6)	80.2–93.9
Regular cleaning and desinfection (*n* = 88)		
Yes	82 (93.2)	43.6–58.9
No	6 (6.8)	2.9–14.4
Waste disposal methods (*n* = 80)		
Burn/bury only	20 (25.0)	16.7–35.6
Sell fertilizer only	9 (11.3)	5.8–20.2
Open dump only	38 (47.5)	36.9–58.3
Open dump and sell as fertilizer	1 (1.3)	0.01–7.4
Burn/bury and sell as fertilizer	9 (11.3)	5.8–20.2
Burn/bury and open dump	1 (1.3)	0.01–7.4
Others	2 (2.5)	0.16–9.2

*n* = number of responses.

**Table 4:** Univariate binary logistic regression analysis of factors associated with levels of biosecurity, AMU, and knowledge among pig farmers in Ogun State.

Variable	Category	Biosecurity			Antimicrobial Use and Practices			Knowledge of AMs and Public Health Consequences		
		OR	95% CI	p-Value	OR	95% CI	p-Value	OR	95% CI	p-Value
Age	<45 >45	-	-	-	-	-	-	1 0.43	0.16–1.15	0.093
Sex	Male Female	1 2.32	0.75–7.20	0.15				3.06	0.82–11.47	0.096
Farm establishment (years)	<6 >6	-	-	-	-	-	-	1 2.16	0.79–5.91	0.133
Existence of a written farm health plan	Yes No	1 0.39	0.16–0.94	0.035 *	-	-	-	-	-	-
Level of education	>Secondary school ≤Secondary school	-	-	-	1 0.18	0.02–1.48	0.111	-	-	-
Use of veterinary services	Yes No	-	-	-	1 0.076	0.02–0.35	0.001 *	-	-	-
Type of production system	Intensive Semi-intensive	-	-	-	-	-	-	1 2.87	1.07–7.74	0.037 *

Key. OR: odds ratio; CI: confidence interval. *: Variables significant at *p* ≤ 0.05. Reference value = 1.

## Supplementary Materials

The following are available online at https://www.mdpi.com/1660-4601/17/10/3579/s1. S1: Signed consent by the Chairperson of the PFAN, Ogun State chapter, to conduct the study among pig production farms in Ogun State. S2: Focus group discussion guidelines/template for pig farmers on perceptions regarding antimicrobial usage and challenges confronting pig production in Ogun State, Nigeria. S3: Questionnaire for the cross-sectional survey—phase 2 of this study.

## Abbreviations

AMR	Antimicrobial resistance
AMs	Antimicrobials
AMSP	Antimicrobial stewardship
AMU	Antimicrobial use
ASF	African swine fever
BLRA	Binary logistic regression analysis
CI	Confidence interval
FAO	Food and Agricultural Organization
FG	Focus group
FGD	Focus group discussion
FMD	Foot and mouth disease
GDP	Gross domestic product
GHSA	Global Health Security Agenda
LGAs	Local government area
OECD	Organization for Economic Co-operation and Development
OIE	World Organization for Animal Health
OR	Odds ratio
PFAN	Pig Farmers’ Association of Nigeria
TADs	Transboundary animal diseases
