# Supplementary material for: Farmers’ Perceptions and Drivers of Antimicrobial Use and Abuse in Commercial Pig Production, Ogun State, Nigeria
Source: Int J Environ Res Public Health. 2020 May 20;17(10):3579. doi: 10.3390/ijerph17103579 (PMC7277550; doi:10.3390/ijerph17103579)
Supplement: Supplementary file 1 [file ijerph-17-03579-s001.pdf]

Supplementary Material 1

Signed consent by the Chairperson, PFAN, Ogun State Chapter to conduct the study among pig production farms in Ogun State.

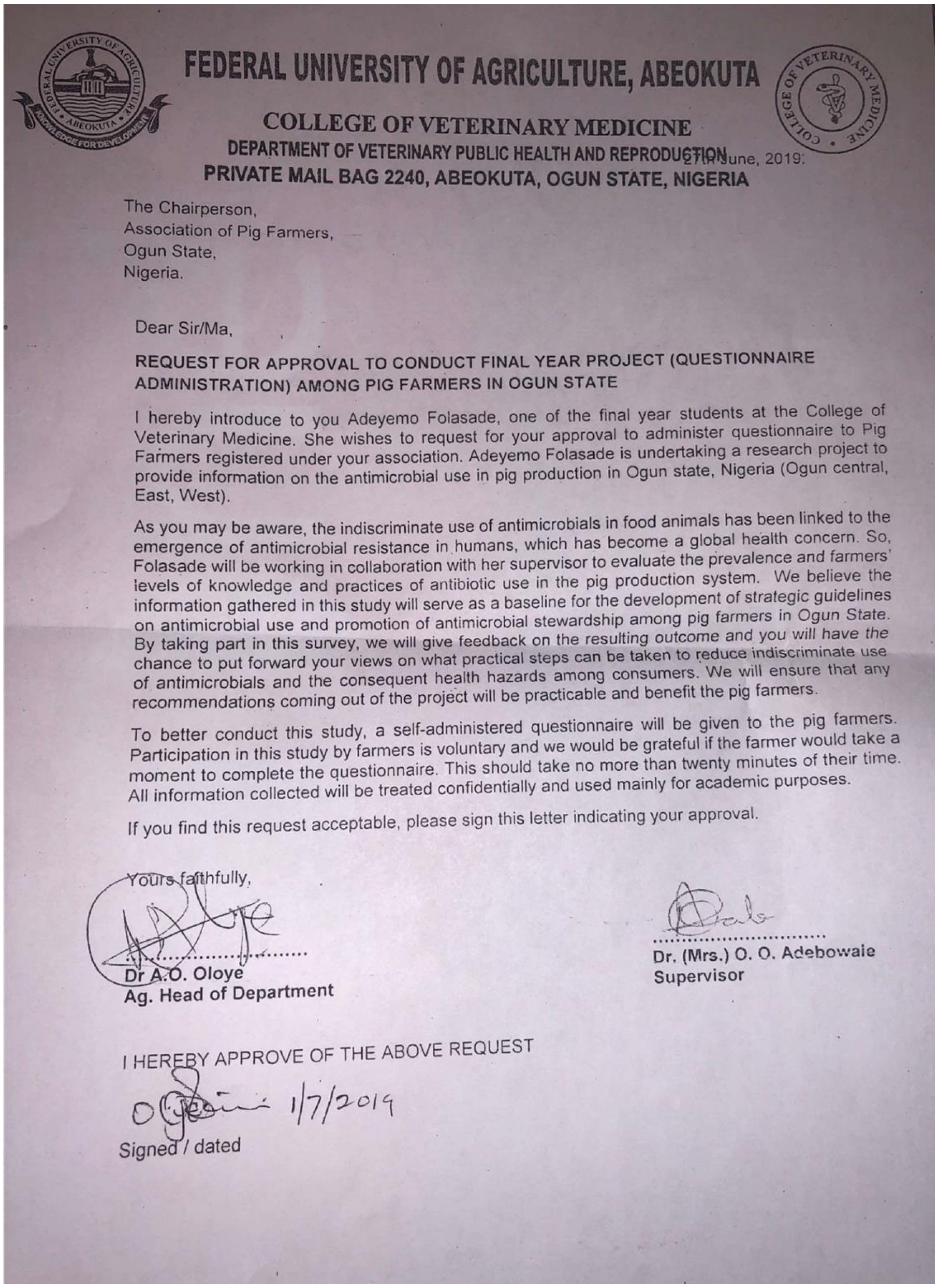

FEDERAL UNIVERSITY OF AGRICULTURE, ABEOKUTA

COLLEGE OF VETERINARY MEDICINE

DEPARTMENT OF VETERINARY PUBLIC HEALTH AND REPRODUCTION

PRIVATE MAIL BAG 2240, ABEOKUTA, OGUN STATE, NIGERIA

27th June, 2019:

The Chairperson,
Association of Pig Farmers,
Ogun State,
Nigeria.

Dear Sir/Ma,

REQUEST FOR APPROVAL TO CONDUCT FINAL YEAR PROJECT (QUESTIONNAIRE ADMINISTRATION) AMONG PIG FARMERS IN OGUN STATE

I hereby introduce to you Adeyemo Folasade, one of the final year students at the College of Veterinary Medicine. She wishes to request for your approval to administer questionnaire to Pig Farmers registered under your association. Adeyemo Folasade is undertaking a research project to provide information on the antimicrobial use in pig production in Ogun state, Nigeria (Ogun central, East, West).

As you may be aware, the indiscriminate use of antimicrobials in food animals has been linked to the emergence of antimicrobial resistance in humans, which has become a global health concern. So, Folasade will be working in collaboration with her supervisor to evaluate the prevalence and farmers' levels of knowledge and practices of antibiotic use in the pig production system. We believe the information gathered in this study will serve as a baseline for the development of strategic guidelines on antimicrobial use and promotion of antimicrobial stewardship among pig farmers in Ogun State. By taking part in this survey, we will give feedback on the resulting outcome and you will have the chance to put forward your views on what practical steps can be taken to reduce indiscriminate use of antimicrobials and the consequent health hazards among consumers. We will ensure that any recommendations coming out of the project will be practicable and benefit the pig farmers.

To better conduct this study, a self-administered questionnaire will be given to the pig farmers. Participation in this study by farmers is voluntary and we would be grateful if the farmer would take a moment to complete the questionnaire. This should take no more than twenty minutes of their time. All information collected will be treated confidentially and used mainly for academic purposes.

If you find this request acceptable, please sign this letter indicating your approval.

Yours faithfully,

....
Dr A.O. Oloye
Ag. Head of Department

....
Dr. (Mrs.) O. O. Adebowale
Supervisor

I HEREBY APPROVE OF THE ABOVE REQUEST

1/7/2019

Signed / dated

Supplementary material 2

Focus group: discussion guidelines/ template for Pig farmers in Ogun State

Title: Perceptions of Farmers Regarding Antimicrobial usage and other Challenges Confronting Pig Production in Ogun State, Nigeria.

Objectives:

1. Facilitator's welcome, a brief introduction about the focus of discussions
2. Provide farmers with the future benefits of the study
3. Perceptions of pig farmers regarding antimicrobial usage and drivers in the pig industry in Ogun state
4. Identify challenges confronting the pig industry in Ogun State?
5. Gather recommendations from pig farmers' perspectives?

Guiding questions:

1. Please, can you identify issues confronting a pig farmer in Ogun state
2. How easy is it to access a food animal veterinarian or services in your area?
3. How well do you have access to veterinary extension officers in your area?
4. Mention common diseases encountered in the pig industry?
5. How do you use antibiotics/AM?
6. What influences your decision to start or discontinue the use of antibiotics/AM?
7. Please share things that are important to you when deciding to use antibiotics/AM.
8. How would antibiotic/AM use affect your production practice?
9. What do you know about antibiotic resistance/AMR?
10. What can producers, veterinarians, and the government do in order to make antibiotic/AM use in pig better?
11. Please share other management practices or products besides antibiotics /AM that you use to prevent or treat disease.
12. In your opinion, what specific type of information would you as pig producers need and like to be receiving about antibiotic use?
13. What is the best format for information dissemination?

Concluding question

14. What is interesting about this topic- AU/AMU?

Conclusion:

Thank the participants

Supplementary Material 3

Questionnaire for the cross-sectional survey- Phase Two of this study

FEDERAL UNIVERSITY OF AGRICULTURE ABEOKUTA

COLLEGE OF VETERINARY MEDICINE

DEPARTMENT OF PUBLIC HEALTH AND REPRODUCTION

PROJECT TITLE: FARM OWNER/MANAGER QUESTIONNAIRE ON FARM OPERATIONS AND ANTIBIOTICS USAGE IN PIG PRODUCTION IN OGUN STATE, NIGERIA.
Farm ID:
Farm name:
Farm Address:
Farm managers name: Date:
Dear Farmer,

I am a final year veterinary student from the Federal University of Agriculture, Abeokuta. I am carrying out a project on the use of antimicrobials among pig farmers.

WHAT IS THE PROJECT ABOUT?

To provide information on the pattern of antimicrobial use (AMU) in pig production in Ogun State, Nigeria with a view to giving insights into the implications of the current status.

Why are we carrying out the project?

As you may be aware, the indiscriminate use of antimicrobials in food animals has been linked to the emergence of antimicrobial resistance in humans, which has become a worldwide health concern due to the associated increased morbidity and mortality in humans

Benefits of the project to farmers?

This study would provide baseline data for efficient development of educational programs and guidelines on AMU among pig farmers, promote strategic planning or initiatives, and policy formulation or modification where appropriate.

What happens to my data?

All information gathered will be treated as highly confidential and will only be used for academic purposes. Please note, the questionnaire should take 15-20 minutes of your time, and is voluntary. Please ensure you tick all relevant boxes and answer the questions appropriately.

Thank You for your time.

SECTION A: FARMERS' SOCIODEMOGRAPHIC DATA

Farm Owner

1. Farm owner's age in years as at last birthday:________________________
2. Sex (Please tick as appropriate): Male ☐ Female ☐
3. Marital status (Please tick as appropriate):

a) Married ☐
b) Single ☐
c) Divorced ☐

4. What is your highest formal educational level?

a) No formal education ☐
b) Primary ☐
c) Secondary ☐
d) Tertiary ☐

Farmers Occupational History

4. Is pig farming your main occupation? Yes ☐ No ☐
5. If No, please specify primary occupation Yes ☐ No ☐
6. If Yes, do you have a secondary occupation ________________________
7. How long have you been operating the farm? ________________________
8. How many hours per day do you work on the pig farm? 1-3hours ☐ 4-6 ☐ >6hours ☐
9. Do you have farm attendants working on your farm? Yes ☐ No ☐
10. If Yes, how many? (please specify number) ________________________

SECTION B: GENERAL FARM INFORMATION

11. Specify farm location (Long, Lat and Altitude) ________________________
(to be filled by visiting vet)
12. Farm size ________________________

Flock details

13. Is pig the animal mainly raised on the farm? Yes ☐ No ☐
14. If Yes, please complete the table below for pigs raised on the farm

Species	Breeds	Source of purchase	Number of males	Number of females	Number/age group of adults	Number/age group of young	Purpose of keep
Pigs							

15. If No, do you operate mixed farming Yes ☐ No ☐

16. Please complete the table below for the other types of animals present on the farm.

Species	Breeds	Source of Purchase	Number of males	Number of females	Number/age group of adults	Number/age group of young	Purpose of keeping
Cattle							
Sheep							
Goat							
Poultry							
Others							

SECTION C: MANAGEMENT AND BIOSECURITY

This section to be filled by farmers for pigs only

Management (please tick as appropriate)

17. What pig production system do you operate? Intensive ☐ semi -intensive ☐
18. Type of housing Open ☐ Close ☐
19. Number of enclosures ______________________
20. Are enclosures bird/other animal-proof? Yes ☐ No ☐
21. Do you have separate enclosures for following?

a) Farrowing Yes ☐ No ☐
b) Weaners Yes ☐ No ☐
c) Growers Yes ☐ No ☐
d) Sows Yes ☐ No ☐
e) Boars Yes ☐ No ☐

22. Are animals in these enclosures permanently held? Yes ☐ No ☐
23. Type of flooring Soil ☐ Concrete ☐ Grass ☐ Gravel ☐

Others: ______________________

24. Type of feed used for the pigs: Industrial byproducts only ☐ Crop residues only ☐ Hotel and party left over ☐ Industrial by product and compounded feed ☐

Biosecurity

25. Does farm owner know about biosecurity? Yes ☐ No ☐
26. Do you use foot dip disinfectant at entrances to farm? Yes ☐ No ☐
27. If Yes, mention disinfectants used:______________________
28. Do you visit other farms within or outside the settlement? Yes ☐ No ☐
29. Do your workers visit within or outside settlement? Yes ☐ No ☐
30. Are pig marketers given access into the farm/pig enclosures? Yes ☐ No ☐
31. Does the feed transport vehicle visit the farm? Yes ☐ No ☐
32. Does the transport vehicle supply feed to other farms? Yes ☐ No ☐

33. Do you have farm own equipment? Yes ☐ No ☐
34. If No, do you get farm equipment from other farms? Yes ☐ No ☐
35. Do you clean and disinfect enclosures? Yes ☐ No ☐
36. Tick waste disposal methods practised on the farm:

Burn/bury	Sell as fertilizer	Dump in refuse site	Others (state)

SECTION D: FARM HEALTH QUESTIONNAIRE

37. Does the farm have isolation facilities for sick animals? Yes ☐ No ☐
38. Are animals that are brought in quarantined before being introduced to the rest of the stock? Yes ☐ No ☐
39. Do you have a routine written health plan? Yes ☐ No ☐
40. Please complete the table below concerning the routine preventive medications used on the unit.

Vaccination	Dewormer	Probiotics	Others(specify)

41. Can you tick below diseases encountered on the farm:

Diseases	Tick
1. African Swine Fever	
2. Salmonellosis	
3. Foot and Mouth diseases	
4. Brucellosis	
5. Coccidiosis	
6. Mycoplasmosis	
7. Helminthiasis	
8. Mastitis	
9. Erysipelas	
10.	

42. Do you keep health records? Yes ☐ No ☐
43. Do you have a farm veterinarian? Yes ☐ No ☐

SECTION E: FARMER'S USE AND PRACTICES OF ANTIBIOTICS USE

44. Have you heard about antibiotics? Yes ☐ No ☐
45. Have you used antibiotics before on your farm Yes ☐ No ☐
46. How often do you use them?
    a. Once in a while ☐
    b. Often ☐
    c. Always ☐
    d. Never ☐
47. Please specify the common antibiotics you use?

Antibiotics	TICK
Tetracycline	
Penicillin	
Amoxicillin	
Streptomycin	
Gentamycin	
Chloramphenicol	
Ciprofloxacin	
Ceftrazone	
Tylosin	
Macrolides	
Polymyxins	
Clarithromycin	
Levofloxacin	
Doxycycline	
Tobramycin	

48. Do you get them off the counter? Yes ☐ No ☐
49. Does a veterinarian prescribe them for use? Yes ☐ No ☐
50. How do you use them?

Prophylactic use	Therapeutic use	Growth promoters	Others (specify)

51. What informs the start of antibiotics?
    a. As soon as you see signs of unwellness ☐
    b. Routine ☐
    c. When the pig is critically sick ☐
    d. Veterinarian prescription ☐
52. What informs the stoppage of antibiotic?
    a. As soon as the animal is showing signs of wellness ☐
    b. Manufacturer's instructions ☐
    c. Veterinarian's advice or prescription ☐

53. What common route of administration do you use?
   a. Orally ☐
   b. Injectable ☐
   c. Suspension ☐
   d. Topically ☐

54. Is the use of antimicrobial influenced by company brand?
55. Do you observe the withdrawal period? Yes ☐ No ☐

SECTION F. FARMERS KNOWLEDGE OF THE USE OF ANTIBIOTICS

56. Do you know antibiotics have different classes? Yes ☐ No ☐
57. Do you know antibiotics have side effects? Yes ☐ No ☐
58. Are you aware that overuse of antibiotics could increase resistance in food-producing animals? Yes ☐ No ☐
59. Are you aware that antibiotic residues from food-producing animals can be passed down to humans through consumption? Yes ☐ No ☐
60. Do you know antimicrobial residues in food animals pose danger to human health? Yes ☐ No ☐
61. Do you know antimicrobial misuse in animals can be linked to resistance in humans? Yes ☐ No ☐
62. Does antimicrobial resistance occur when bacteria develop the ability to defeat the drugs designed to kill them? Yes ☐ No ☐
63. Does resistance increase morbidity and mortality in animals? Yes ☐ No ☐
64. Does resistance increase morbidity and mortality in humans? Yes ☐ No ☐
65. Does antimicrobial resistance increase cost of production? Yes ☐ No ☐
66. Is it safe not to complete the dose of antibiotics? Yes ☐ No ☐
67. Is antimicrobial sensitivity test necessary before use? Yes ☐ No ☐
68. Are you aware of antimicrobial stewardship? Yes ☐ No ☐
69. What do you understand by the words antimicrobial stewardship…**………………...**
